# Decreased synaptic vesicle glycoprotein 2A binding in a rodent model of familial Alzheimer's disease detected by [^18^F]SDM-16

**DOI:** 10.3389/fneur.2023.1045644

**Published:** 2023-02-08

**Authors:** Chao Zheng, Takuya Toyonaga, Baosheng Chen, LaShae Nicholson, William Mennie, Michael Liu, Joshua Spurrier, Kristin Deluca, Stephen M. Strittmatter, Richard E. Carson, Yiyun Huang, Zhengxin Cai

**Affiliations:** ^1^Department of Radiology and Biomedical Imaging, PET Center, Yale School of Medicine, New Haven, CT, United States; ^2^Program in Cellular Neuroscience, Neurodegeneration, and Repair, Departments of Neuroscience and Neurology, Yale University School of Medicine, New Haven, CT, United States

**Keywords:** [^18^F]SDM-16, APP/PS1, SV2A, PET, brain, SRTM, DVR, SUVR

## Abstract

**Introduction:**

Synapse loss is one of the hallmarks of Alzheimer's disease (AD) and is associated with cognitive decline. In this study, we tested [^18^F]SDM-16, a novel metabolically stable SV2A PET imaging probe, in the transgenic APPswe/PS1dE9 (APP/PS1) mouse model of AD and age-matched wild-type (WT) mice at 12 months of age.

**Methods:**

Based on previous preclinical PET imaging studies using [^11^C]UCB-J and [^18^F]SynVesT-1 in the same strain animals, we used the simplified reference tissue model (SRTM), with brain stem as the pseudo reference region to calculate distribution volume ratios (DVRs).

**Results:**

To simplify and streamline the quantitative analysis, we compared the standardized uptake value ratios (SUVRs) from different imaging windows to DVRs and found that the averaged SUVRs from 60–90 min post-injection (*p.i*.) are most consistent with the DVRs. Thus, we used averaged SUVRs from 60–90 min for group comparisons and found statistically significant differences in the tracer uptake in different brain regions, e.g., hippocampus (*p* = 0.001), striatum (*p* = 0.002), thalamus (*p* = 0.003), and cingulate cortex (*p* = 0.0003).

**Conclusions:**

In conclusion, [^18^F]SDM-16 was used to detect decreased SV2A levels in the brain of APP/PS1 AD mouse model at one year old. Our data suggest that [^18^F]SDM-16 has similar statistical power in detecting the synapse loss in APP/PS1 mice as [^11^C]UCB-J and [^18^F]SynVesT-1, albeit later imaging window (60–90 min *p.i*.) is needed when SUVR is used as a surrogate for DVR for [^18^F]SDM-16 due to its slower brain kinetics.

## 1. Introduction

Synaptic loss is considered as one of the most robust and consistent neuropathological biomarkers of Alzheimer's disease (AD). The loss of synapses and several presynaptic proteins is observed at the earliest stages of AD (1). Synaptic vesicle glycoprotein 2A (SV2A) is an essential protein expressed in all presynaptic terminals and is involved in the regulation of synaptic exocytosis and endocytosis (2). Thus, SV2A positron emission tomography (PET) tracers may be used to measure changes of synaptic density, and facilitate early AD diagnosis (3) and the development of AD treatments at preclinical and clinical stages (4–6). With the lead SV2A PET tracer, [^11^C]UCB-J (7), the preclinical and clinical quantitative assessment of SV2A changes in AD has been achieved. Compared to control subjects, statistically significant reduction of SV2A in the hippocampus of AD patients was identified (8, 9). [^18^F]UCB-H was also used to evaluate patients with mild cognitive impairment (MCI) or AD (10). In addition to studying AD pathophysiology, repeated [^11^C]UCB-J PET was used to monitor the treatment effects of saracatinib in the APP/PS1 mouse model of familial AD (11). For example, [^11^C]UCB-J binding was shown to be 9.8% lower in the hippocampus of APP/PS1 mice than WT mice (11). Similarly, hippocampal binding of [^18^F]SynVesT-1, a newer ^18^F-labeled SV2A tracer, was found to be 7.4% less in APP/PS1 mice than their littermate controls (12, 13). Recently, we synthesized and evaluated [^18^F]SDM-16, a novel SV2A PET tracer with high binding affinity and metabolic stability in non-human primates (14, 15). The objective of this study is to assess the brain kinetics of [^18^F]SDM-16 in mice and identify a simplified method for its quantification in APP/PS1 and control mice. We also compared the performance of [^18^F]SDM-16 in detecting synapse loss in APP/PS1 mice with that of [^11^C]UCB-J and [^18^F]SynVesT-1. *In vivo* assessments of [^18^F]SDM16 binding to mouse SV2A is an important validation step toward its application in mouse disease models to investigate synaptic density dynamics following drug treatment or other interventions.

## 2. Materials and methods

### 2.1. Radiochemistry

Instrumentation for radiochemistry procedures and production of (*R*)-4-(3-fluoro-5-(fluoro-^18^F)phenyl)-1-((2-methyl-1*H*-imidazol-1-yl)methyl)pyrrolidin-2-one ([^18^F]SDM-16) have been described previously (15). In brief, radiosynthesis of [^18^F]SDM-16 was achieved *via* copper (II) catalyzed reaction of the corresponding tin precursor, (*R*)-4-(3-fluoro-5-(trimethylstannyl)phenyl)-1-((2-methyl-1*H*-imidazol-1-yl)methyl)pyrrolidin-2-one, with ^18^F^−^ in the presence of pyridine and potassium carbonate. Chemical purity, radiochemical purity, and molar activity were determined by high-performance liquid chromatography (HPLC) analysis of the final product solution. Identity of the labeled compound was confirmed by co-injection of the product solution with the unlabeled reference standard.

### 2.2. Animals and PET imaging experimental set-up

All animal experiments were approved by the Yale Institutional Animal Care and Use Committee for compliance with National Institutes of Health requirements on the use of laboratory animals. WT C57/B6J mice and amyloid precursor protein and presenilin 1 double-transgenic [APPswe/PS1*D*E9 (APP/PS1)] mice were purchased from Jackson Laboratories and maintained on a C57/B16J background as described previously (16). For PET scanning, mice (53 ± 2 weeks) were kept on a heating pad and anesthetized with 0.75~2.5 % isoflurane. Mice were administered with 150 μL of [^18^F]SDM-16 (8.9 ± 6.7 MBq, molar activity of 286 GBq/μmol at the end of synthesis, *n* = 2) formulated with saline containing <10% *v*/*v* EtOH, *via* intravenous (*i.v*.) tail-vein injection. Dynamic PET data were acquired on the Siemens Focus 220 scanners for a total of 90 min post-radiotracer injection (*p.i*., *n* = 9 per group).

### 2.3. Image analysis

PET images were reconstructed with 3D ordered subset expectation maximization method (OSEM3D; 2 iterations, 16 subsets) with maximum a posteriori probability algorithm (MAP; 25 iterations). Corrections for decay, attenuation, scatter, normalization and randoms were applied. An empirically determined system calibration factor (in units of Bq/cps) combined with the decay corrected administered activity and the animals' weights were used to calculate the standardized uptake value (SUV). An averaged PET image for each measurement (mean of all frames) was co-registered to a representative PET image of [^18^F]SDM-16 resliced in the Ma-Benveniste-Mirrione mouse brain atlas space. Registration was performed with a 6 degree-of-freedom linear registration using FMRIB's Linear Image Registration Tool in FSL. Regions of interest (ROIs) were extracted from the brain atlas and regional time activity curves (TACs) were obtained by applying template ROIs to the PET images. The following ROIs were included: brain stem (BS), cerebellum (CB), cingulate cortex (CCX), cortex (CX), hippocampus (HC), inferior colliculi (IC), mid brain (MB), striatum (ST), superior colliculi (SC), thalamus (TH), and whole brain (WB).

### 2.4. Quantitative analysis

In a cohort of three WT and three APP/PS1 mice, the simplified reference tissue model (SRTM) (17, 18) was used to estimate distribution volume ratios (DVRs), using 90 min dynamic PET scan data and brain stem (BS) as the pseudo reference region. SUVs of brain regions averaged from 40 to 70, 50 to 80, and 60 to 90 min *p.i*. were normalized with BS to generate SUV ratios (SUVRs). SUVRs from different time windows were correlated with DVRs to determine the optimal static imaging window. The SUVRs from the optimal imaging window was used in subsequent data analysis.

### 2.5. Statistical analysis

The primary target region was the hippocampus. All variables are presented as mean ± SD. For group differences, unpaired *t* tests were applied. *P* < 0.05 without correction for multiple comparisons were considered statistically significant. Sample sizes were analyzed by G^*^Power, a free statistical program for power analysis (http://www.gpower.hhu.de/en.html; Heinrich Heine University, Düsseldorf, Germany software) (19).

## 3. Results

### 3.1. Radiochemistry

The SV2A PET tracer [^18^F]SDM-16 was synthesized using its enantiopure trimethyltin precursor following published procedures (15), with >99% radiochemical and enantiomeric purity, as determined by reverse phase C18 and chiral HPLC analysis. Molar activity at the end of synthesis (EOS) was 286 GBq/μmol (*n* = 2). Total synthesis time including purification and formulation was around 90 min.

### 3.2. Rodent PET imaging and data analysis

**Figure 1:**
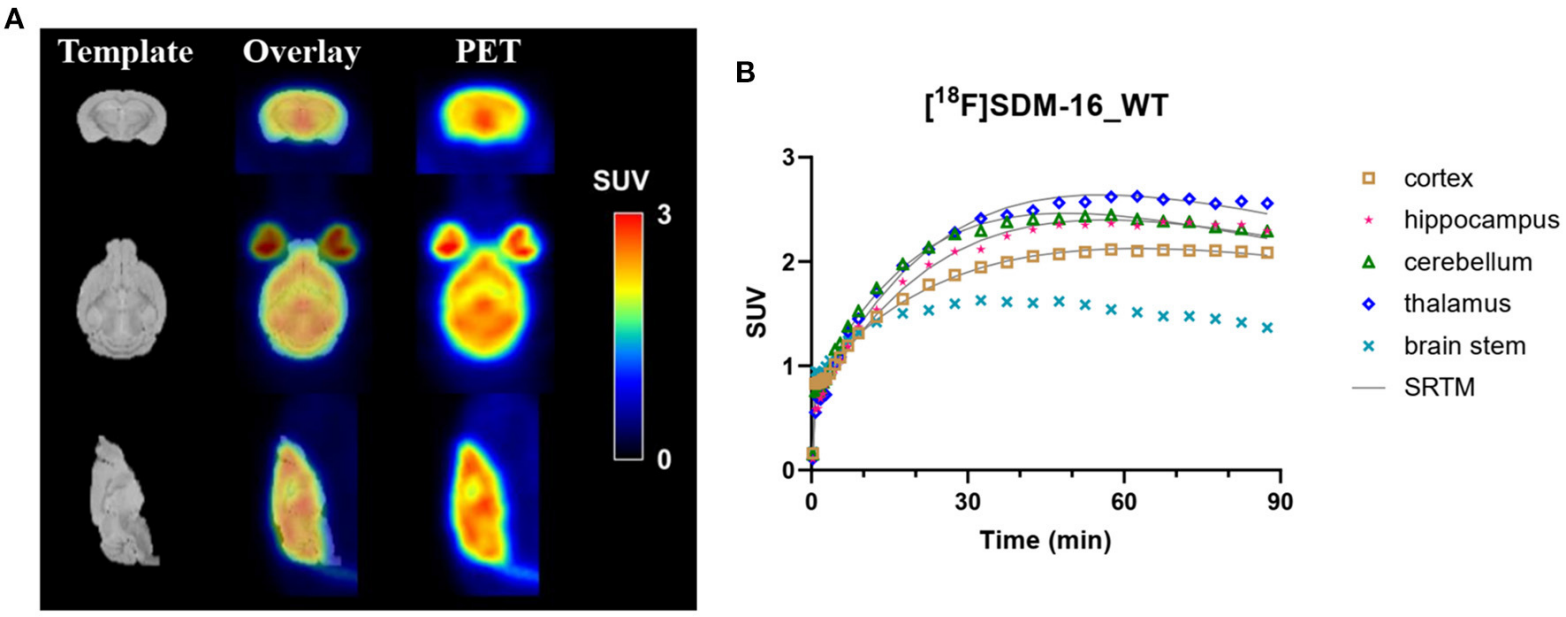
**(A)** Representative summed SUV (0–60 min) images of [^18^F]SDM-16 in a wild-type (WT) mouse brain and the overlaid images with an MR template. **(B)** The time-activity curves of [^18^F]SDM-16 in selected brain regions (cortex, hippocampus, cerebellum, thalamus, and brain stem) of a WT mouse. Solid lines are SRTM-fitted curves, using brain stem as the reference region.

The rodent brain PET images showed ubiquitous [^18^F]SDM-16 uptake in gray matter (Figure 1A). Representative TACs of [^18^F]SDM-16 in a WT mouse brain were shown in Figure 1B. The TACs indicated efficient brain penetration (SUV up to 2.4 after 30 min *p.i*.), and a steady increase in uptake over 30–60 min *p.i*., followed by a relatively slow washout phase. The highest uptake was in the inferior colliculi among the brain regions analyzed, and the lowest uptake in the BS, which is consistent with our previous results using [^11^C]UCB-J and [^18^F]SynVesT-1 (12). Therefore, we chose BS as the pseudo reference region in the SRTM analysis. Satisfyingly, the TACs were well-described by SRTM (Figure 1B). The regional SUVRs of [^18^F]SDM-16 correlated well with those of [^11^C]UCB-J and [^18^F]SynVesT-1 (Supplementary Figure 1, R^2^ = 0.66, *p* < 0.0001 with [^11^C]UCB-J; R^2^ = 0.68, *p* < 0.0001 with [^18^F]SynVesT-1).

**Figure 2:**
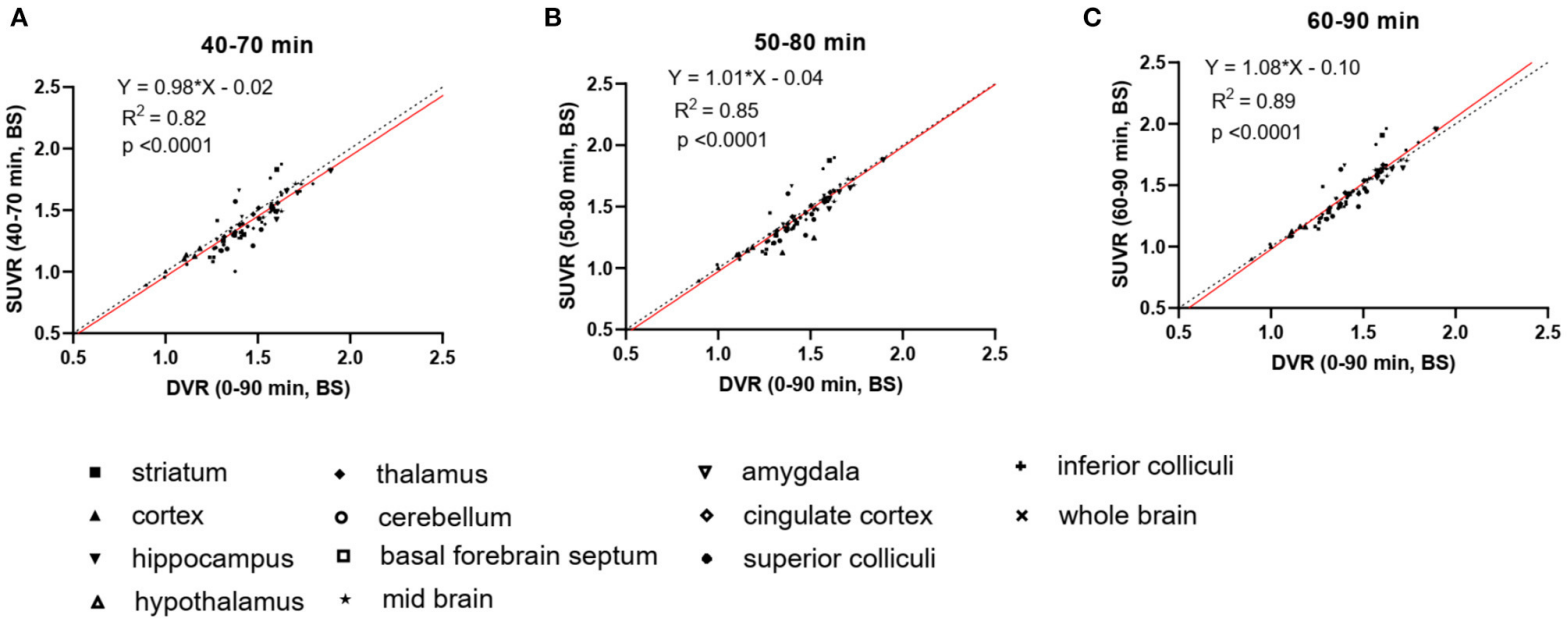
Linear regression analysis of DVR and SUVR using averaged SUVs from three different imaging windows, i.e., **(A)** 40–70 min p.i., **(B)** 50–80 min p.i., and **(C)** 60–90 min p.i. DVRs are calculated through SRTM using 90-min dynamic scan data and brain stem as reference region, *N* = 3 for each genotype. Dashed lines are lines of identity.

To increase the throughput of PET scanning and simplify the quantification method, we compared the averaged SUVRs from different imaging windows (40–70, 50–80, and 60–90 min) with the DVRs calculated using the complete 90 min data set to identify a suitable static imaging window for [^18^F]SDM-16 in mouse brain PET (Figure 2). The SUVRs at all time intervals correlated well with DVRs (R^2^ = 0.82, 0.85, and 0.89, for imaging windows 40–70, 50–80, and 60–90 min, respectively). The averaged SUVRs from 60 to 90 min *p.i*. were most consistent with the DVRs (Figure 2C, Y = 1.08^*^X−0.10, R^2^ = 0.89, *p* < 0.0001), compared with the other imaging windows. As expected, the SUVRs from earlier time windows underestimated the DVRs. The percentage differences between SUVR and DVR decreased from 5.2 ± 3.3% (40–70 min) and 4.2 ± 2.8% (50–80 min) to 3.8 ± 2.4% (60–90 min) (Supplementary Table 1). Therefore, the 60–90 min *p.i*. was identified as a suitable static imaging window to allow for the use of SUVR as a surrogate of DVR for [^18^F]SDM-16 in the mouse brain.

**Figure 3:**
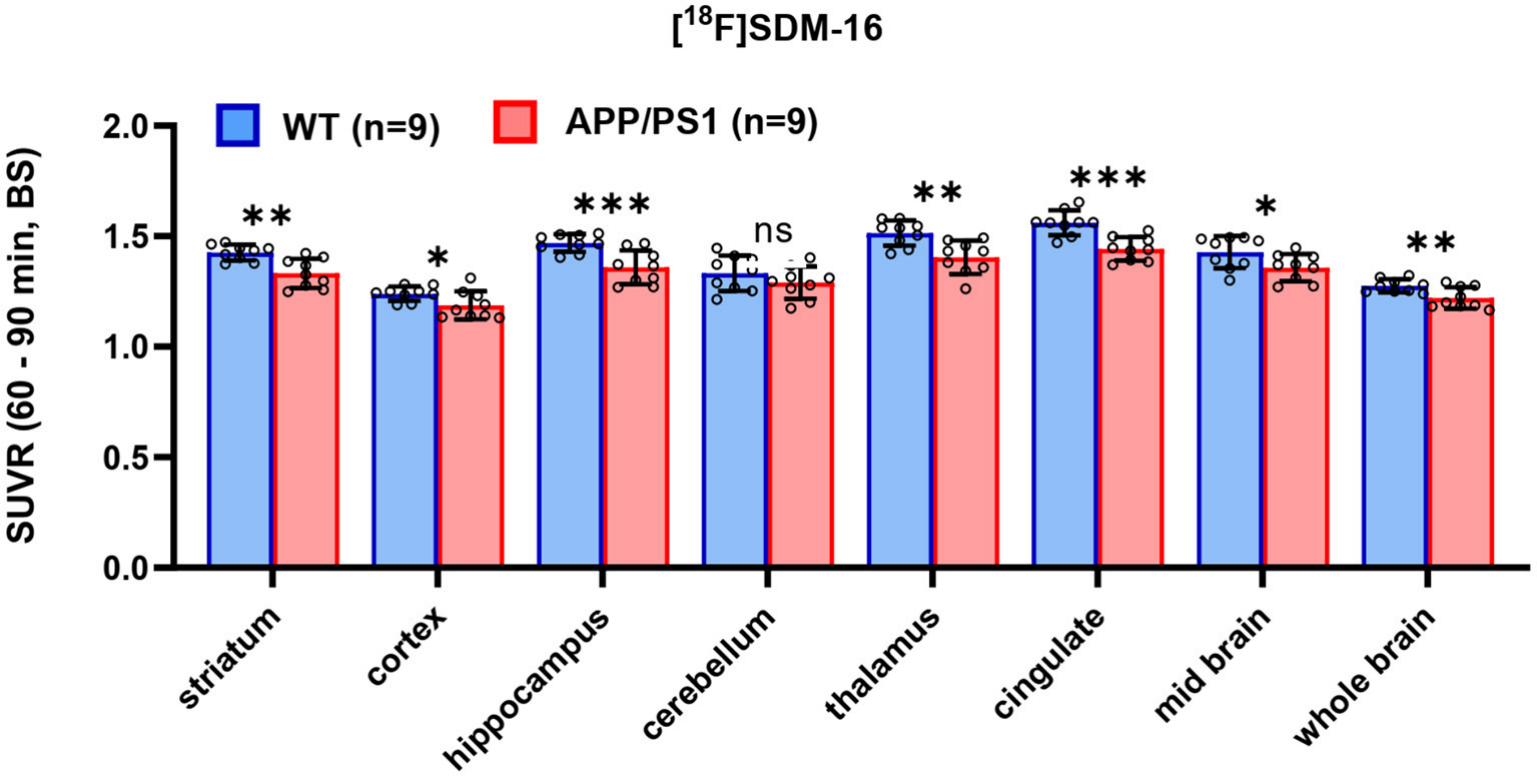
Comparison of SUVR in selected brain regions for WT and APP/PS1 mice (*n* = 9 for each group, ***p < 0.001; **p < 0.01; 0.01 < ^*^p ≤ 0.05; ns, statistically non-significant).

After identifying the best static imaging time window, we compared the SUVR (60–90 min) of [^18^F]SDM-16 in APP/PS1 and WT mice. The whole brain SUVR (60–90 min) was 4.3% lower in 12-month-old APP/PS1 mice (*n* = 9) than in age-matched WT controls (*n* = 9). The most profound differences were found in hippocampus (7.5%, *p* = 0.001), cingulate cortex (7.6%, *p* = 0.0003), thalamus (7.2%, *p* = 0.003), followed by striatum (6.6%, *p* = 0.001), mid brain (4.9%, *p* = 0.04), cortex (4.2%, *p* = 0.04), and cerebellum (3.2%, *p* = 0.2) (Figure 3 and Supplementary Table 2). These results are consistent with previously published SUVR (30–60 min) results using [^11^C]UCB-J and [^18^F]SynVesT-1. With [^11^C]UCB-J (*n* = 9), the averaged whole brain SUVR (30–60 min) in APP/PS1 mice was 6.9% (*p* = 0.015) lower than age-matched WT controls, with the most profound differences found in hippocampus (9.8%, *p* = 0.017), thalamus 9.6%, *p* = 0.028), striatum (9.1%, *p* = 0.023), and cingulate cortex (6.8%, *p* = 0.024) (Supplementary Figure 2 and Supplementary Table 3). With [^18^F]SynVesT-1, the averaged whole brain SUVR (30–60 min) was 3.5% lower in 12-month-old APP/PS1 mice (*n* = 24) than age-matched WT controls. The most profound differences were found in hippocampus (7.4%, *p* < 0.0001), followed by thalamus (5.5%, *p* < 0.0001), cingulate cortex (5.1%, *p* < 0.0001), striatum (4.4%, *p* = 0.004), and cortex (3.6%, *p* = 0.012) (Supplementary Figure 2 and Supplementary Table 4).

**Figure 4:**
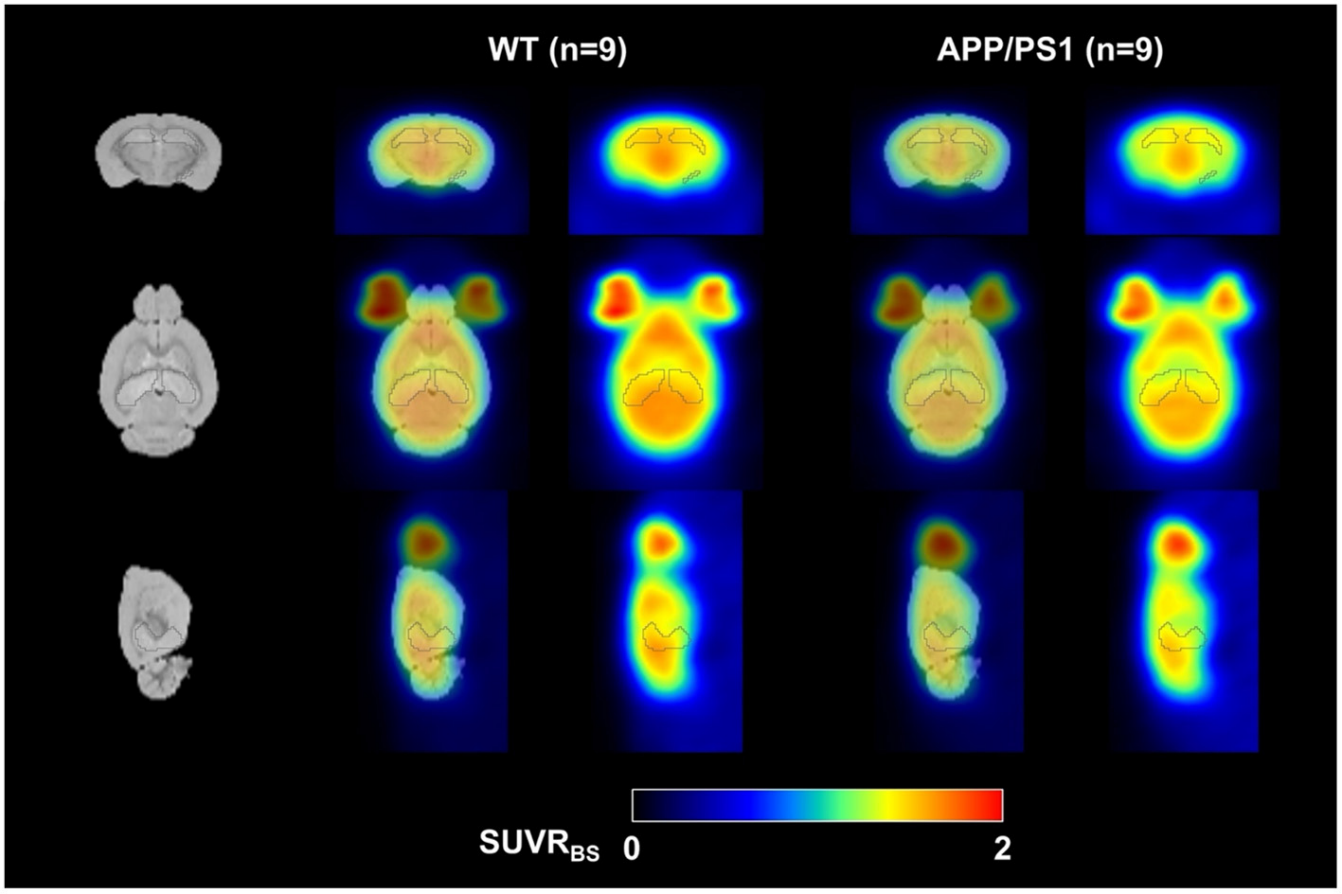
Averaged [^18^F]SDM-16 SUVR images for WT (*n* = 9) and APP/PS1 (*n* = 9) mouse brains. Color scale represents SUVR, averaged from 60 to 90 min p.i. and normalized to brain stem.

In this study, the injected mass of SDM-16 was (0.55 ± 0.75) μg/kg, with no significant group difference (Supplementary Figure 3A, *p* = 0.98). We do not expect to see any significant mass effect on binding, as there is no statistically meaningful correlation between the brain SUVs and the administered tracer mass (Supplementary Figure 3B, *p* = 0.24 for hippocampus; *p* = 0.27 for whole brain). Voxel-based parametric statistical analysis also showed a global decrease in tracer uptake in the APP/PS1 mouse brain, with the most significant decrease in hippocampus, which is consistent with the results from ROI-based analysis (Figure 4).

### 3.3. Results of the power analysis

**Figure 5:**
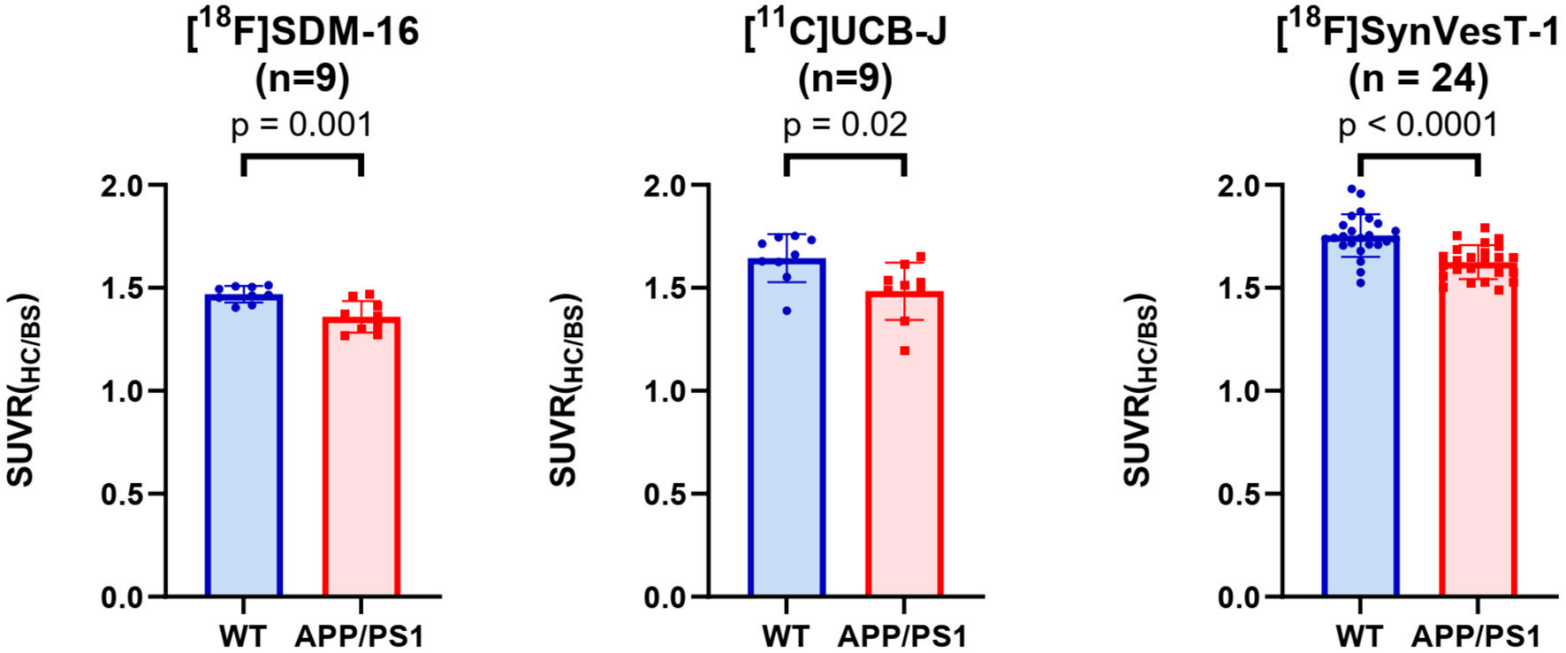
Group comparisons of hippocampal SUVRs in APP/PS1 mice and WT mice, using [^18^F]SDM-16, [^11^C]UCB-J, and [^18^F]SynVesT-1 and the corresponding optimal imaging windows, i.e., 60–90, 30–60, and 30–60 min, for [^18^F]SDM-16, [^11^C]UCB-J and [^18^F]SynVesT-1, respectively.

Because the synapse loss in hippocampus is the most significant in APP/PS1 mice (Figure 5), we used the G^*^Power to estimate the reasonable sample sizes for different SV2A radioligands at predefined statistical power levels (Table 1). In context, studies with [^11^C]UCB-J require sample sizes of 13 (power = 0.9), and 9 (power = 0.8) for a one-tailed test. For a two-tailed test, sample sizes of 15 (power = 0.9) and 12 (power = 0.8) were required for [^11^C]UCB-J. For studies using [^18^F]SynVesT-1, we found sample size requirements dropped to 10 (power = 0.9) and 7 (power = 0.8) for a one-tailed test, 12 (power = 0.9) and 9 (power = 0.8) for a two-tailed test.

**Table 1:** Power analysis based on hippocampal SUVR of [^18^F]SDM-16 (60–90 min), [^11^C]UCB-J (30–60 min), and [^18^F]SynVesT-1 (30–60 min) in APP/PS1 and WT mice.

	**SUVR**			***N*** **number (one-tail)**		***N*** **number (two-tail)**	
**Tracer**	**WT (*****n*****, Mean** ±**SD)**	**APP/PS1 (*****n*****, Mean** ±**SD)**	**Mean difference**	**80% power**	**90% power**	**80% power**	**90% power**
[^18^F]SDM-16	9, 1.47 ± 0.04	9, 1.36 ± 0.08	0.11	5	7	7	9
[^11^C]UCB-J	9, 1.64 ± 0.12	9, 1.48 ± 0.14	0.16	9	13	12	15
[^18^F]SynVesT-1	24, 1.75 ± 0.10	24, 1.62 ± 0.08	0.13	7	10	9	12

The required sample sizes per genotype were reduced even further for [^18^F]SDM-16: *n* = 7 (power = 0.9), and *n* = 5 (power = 0.8) for one-tailed test; *n* = 9 (power = 0.9) and n = 7 (power = 0.8) for a two-tailed test. This finding indicated that studies with [^18^F]SDM-16 requires less animals to achieve the same statistical power for future experiments. Under two-tailed (power = 0.9) conditions, the reasonable sample sizes were calculated for other brain regions for [^18^F]SDM-16, [^11^C]UCB-J, and [^18^F]SynVesT-1 (Supplementary Table 5).

## 4. Discussion

Synapse loss is a robust pathology in AD (20). Non-invasive measurement of synaptic density provided a method to track synaptic alterations *in vivo* during the AD pathogenesis, and to objectively monitor therapeutic effects. As a sensitive and quantitative imaging modality, SV2A PET has been used in clinical neuroimaging studies of a variety of neuropsychiatric disorders (5). As new interventions are emerging to treat AD at different stages (21), the application of SV2A PET at the preclinical phase will facilitate the evaluations of these new treatments in relevant animal models. However, the accurate quantification of synapse loss in small rodent brains poses seemingly formidable challenges, mainly due to the intrinsic spatial resolution of small animal PET scanners and the partial volume effects (PVE) (22). Previous work from our group proved for the first time that it is possible to detect synapse loss in the hippocampus of APP/PS1 mice relative to their WT controls using [^11^C]UCB-J (11). Glorie et al. performed longitudinal SV2A PET studies and detected synaptic density changes in the brain of a transgenic animal model of obsessive-compulsive disorder using [^11^C]UCB-J (23). Decreases of 2.5 and 5.7% in [^11^C]UCB-J binding was detected in the ipsilateral striatum of rats at 15- and 22-weeks post injection of alpha synuclein fibrils, respectively (24). Using [^18^F]UCB-H, progressive synapse loss was detected in a rat model of epilepsy (25, 26). Our group evaluated [^18^F]SynVesT-1, an ^18^F-labeled 3,5-difluorobenzyl analog of UCB-J, in APP/PS1 mice and simplified the quantification method by using SUVR from 30 to 60 min *p.i*. (12). [^18^F]SynVesT-1 turned out to be comparable to [^11^C]UCB-J in terms of brain kinetics and specific binding signals in mouse brain, but more practical to use in rodent PET imaging studies due to the longer half-life of ^18^F (110 min) than ^11^C (20 min). To image the synapse dynamics beyond the brain and in the whole CNS (27), we recently developed [^18^F]SDM-16 as an SV2A ligand with improved binding affinity and metabolic stability, which showed high specific binding signals in non-human primate brains, with relatively slow kinetics. Interestingly, in mouse brains, we observed lower uptake and faster kinetics for [^18^F]SDM-16 than seen in non-human primates (Figure 1). However, in the same species, the kinetics of [^18^F]SDM-16 remains slower than that of [^11^C]UCB-J and [^18^F]SynVesT-1.

Although the apparent kinetics of [^18^F]SDM-16 in the non-human primate brain is relatively slow, the time-activity curves are well-described with the simple one-tissue compartment (1TC) model, which produced volume of distribution values reliably with low standard errors (15). Due to the lack of arterial input function in the mouse PET imaging study, we applied SRTM to calculate the DVR in selected brain subregions, with brain stem as the pseudo reference region, because there is no absolute reference region for SV2A PET data analysis (28) and the SV2A PET tracers uptake in the brain stem appears to be the lowest among all the ROIs. As SRTM assumes 1TC characteristics of the kinetics in the target brain regions, we performed reference Logan analysis to check the 1TC nature of the kinetics and cross-validated the DVRs estimated by both methods (Supplementary Figure 4). We found that the reference Logan derived DVRs (t^*^ = 0 min) are consistent with DVRs estimated with t^*^ = 10 min (Supplementary Figure 4A, Y = 0.80×X + 0.20, R^2^ = 0.95). Also, the reference Logan DVRs correlated well with SRTM-derived DVRs (Supplementary Figure 4B, Y = 0.94×X−0.10, R^2^ = 0.95) and SUVR (60–90 min) (Supplementary Figure 4C, Y = 1.21×X−0.13, R^2^ = 1.00).

In previous studies using [^11^C]UCB-J and [^18^F]SynVesT-1 in the same rodent model (APP/PS1 and C57BL/6J littermate control), BS was used as a pseudo reference region to calculate the binding potential (*BP*_ND_), and SUVR-1 as a surrogate for *BP*_ND_. Presumably, because of the better signal statistics of [^18^F]SynVesT-1, BS showed lower variability in our rodent PET imaging study (12). The optimal static imaging window for [^11^C]UCB-J and [^18^F]SynVesT-1 is from 30 to 60 min *p.i*. As expected, the appropriate static imaging window for [^18^F]SDM-16 in mouse brain (60–90 min *p.i*. in Figure 2) is later than that of [^11^C]UCB-J and [^18^F]SynVesT-1 (30–60 min *p.i*.), reflecting its slower brain kinetics than [^11^C]UCB-J and [^18^F]SynVesT-1.

As there is likely a certain extent of synapse loss in BS of APP/PS1 mice (29, 30), we are likely underestimating the group differences by using BS as the pseudo reference region. We did not observe any statistically significant difference in cerebellum (*p* = 0.25) (Figure 3 and Supplementary Table 2), which suggests that the changes in SV2A level in cerebellum is similar to BS in 1-year-old APP/PS1 mice. The SPM analysis showed global decrease in tracer uptake in the APP/PS1 mice, with the most significant decrease in the hippocampus, corroborating the ROI-based analysis. These imaging results are consistent with previous histological staining analysis of SV2A and PSD-95 in the dentate gyrus and cortex of 1-year old APP/PS1 mice (13), where a 24% decrease in SV2A expression was observed in dentate gyrus, and a 20% decrease in cortex. The relatively lesser group differences we observed in SV2A PET could be due to partial volume effects in small animal PET imaging as discussed in a recent review article (31).

Using SUVR as the readout, we performed statistical power analysis for [^18^F]SDM-16, [^11^C]UCB-J, and [^18^F]SynVesT-1 (Table 1) and found that these tracers share similar effect sizes and sample sizes to achieve the same level of statistical power to detect the synapse loss in the hippocampi of APP/PS1 mice, though [^18^F]SDM-16 requires slightly smaller sample sizes (Table 1). This is due to the slightly lower variability in [^18^F]SDM-16 SUVRs. Nonetheless, it should be noted that these comparisons were based on results from studies using different cohorts of animals.

## 5. Conclusions

In summary, we demonstrated that the radioligand [^18^F]SDM-16 had high uptake in mouse brain, and APP/PS1 mice showed lower tracer uptake than WT mice, supporting the use of [^18^F]SDM-16 in preclinical AD drug discovery and development. We applied a simplified quantification method in APP/PS1 mice and WT controls using [^18^F]SDM16 and small animal PET, supporting further evaluation and validation of this tracer in other disease models. SUVR from 60 to 90 min *p.i*. using BS as reference region provides reliable estimation of DVR. Using this simplified quantification method, [^18^F]SDM16 could be used to estimate SV2A levels in rodent models, providing a translational method for tracking disease progression and testing treatment effects.

## Data availability statement

The original contributions presented in the study are included in the article/Supplementary material, further inquiries can be directed to the corresponding author.

## Ethics statement

The animal study was reviewed and approved by Yale Institutional Animal Care and Use Committee.

## Author contributions

CZ, BC, and ZC designed research. CZ synthesized the radiotracer and wrote the manuscript. CZ, TT, BC, LN, ML, WM, and JS conducted research and oversaw collection of imaging data. CZ and TT analyzed imaging data. KD arranged experiment animals. CZ, ZC, SS, RC, and YH edited the final manuscript. All authors have read and approved the final manuscript.
