# Supplementary material for: Decreased synaptic vesicle glycoprotein 2A binding in a rodent model of familial Alzheimer's disease detected by [18F]SDM-16
Source: Front Neurol. 2023 Feb 8;14:1045644. doi: 10.3389/fneur.2023.1045644 (PMC9945093; doi:10.3389/fneur.2023.1045644)
Supplement: Supplementary file 1 [file Table_1.DOCX]

**Supplementary material**

**Fig. S1**. Linear regression analysis of SUVR (60-90 min, region-to-brain stem) of [^18^F]SDM-16 with SUVR (30-60 min, region-to-brain stem) of [^11^C]UCB-J and [^18^F]SynVesT-1, respectively. Dashed lines are lines of identity.

**Fig. S2.** Comparison of SUVR_(BS)_ of WT and APP/PS1 mice in brain subregions using [^18^F]SDM-16, [^11^C]UCB-J and [^18^F]SynVesT-1 (*****p* <0.0001; ****p* <0.001; ***p* <0.01; 0.01< **p* ≤ 0.05. ns, not significant).

(**A**)

(**B**)

**Fig. S3. (A)** The injected mass of SDM-16 in WT (blue dots) and APP/PS1 (red dots) mice. **(B)** Lack of correlation between injected mass of SDM-16 with [^18^F]SDM-16 SUV in hippocampus (HC) and whole brain (WB).

(**B**)

(**A**)

(**C**)

**Fig. S4.** (**A**) Linear correlation between reference Logan-derived DVRs using t* = 10 min (x-axis) and those using t* = 0 min (y-axis); (**B**) Correlation between DVRs estimated with reference Logan (t* = 0) against DVRs estimated with SRTM; (**C**) Linear correlation between SUVR (60-90 min) and DVR (reference Logan, t* = 0). Brain stem (BS) is used as the reference region. n = 3. Dashed lines are lines of identity.


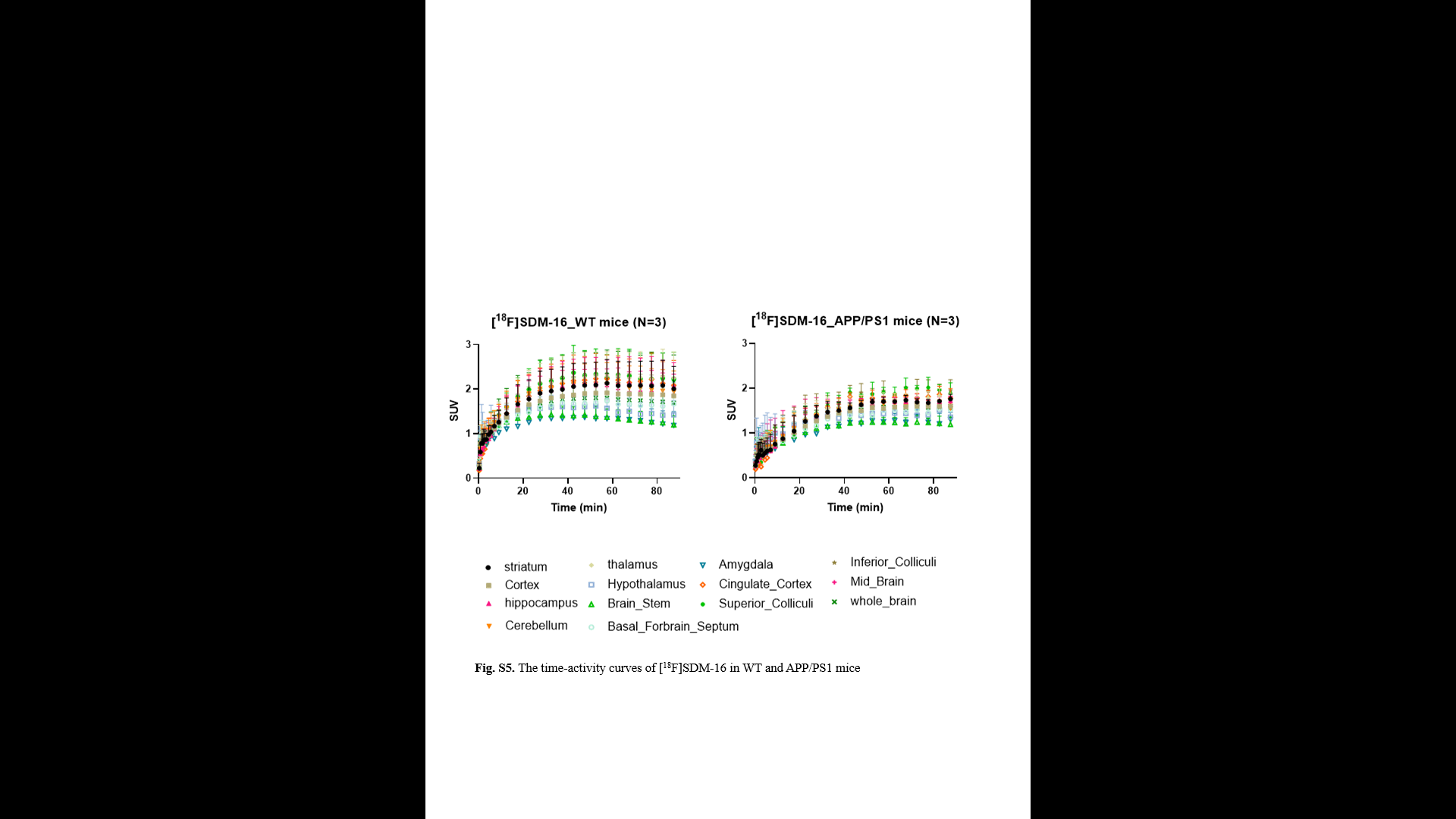


**Fig. S5**. The time-activity curves of [^18^F]SDM-16 in WT and APP/PS1 mice.

**Table S1:** Percentage differences of SUVRs at three different time windows (40-70 min, 50-80 min, and 60-90 min, p.i.) with DVRs of [^18^F]SDM-16 in different brain regions (n = 3). Percentage difference = (DVR - SUVR)/DVR × 100%.

| Brain regions | Percentage difference (mean ± SD) | | |
| --- | --- | --- | --- |
|  | 40-70 min | 50-80 min | 60-90 min |
| Striatum | 7.8 ± 3.2% | 6.2 ± 4.6% | 4.5 ± 6.3% |
| Cortex | 11.6 ± 4.2% | 9.8 ± 4.5% | 7.9 ± 5.7% |
| Hippocampus | 6.7 ± 4.1% | 5.2 ± 5.9% | 4.6 ± 7.1% |
| Thalamus | 7 ± 5% | 4.7 ± 6.4% | 5.6 ± 7.6% |
| Cerebellum | 4.9 ± 6.9% | 4 ± 7.6% | 5 ± 6.9% |
| Basal forebrain septum | 10.3 ± 2.9% | 9.1 ± 2.7% | 7.7 ± 4.8% |
| Amygdala | 3.2 ± 2.1% | 2.4 ± 2.1% | 1.8 ± 1.4% |
| Cingulate cortex | 4.2 ± 4.2% | 2.3 ± 2.1% | 1.4 ± 1.4% |
| Superior colliculi | 4.1 ± 3% | 2.3 ± 2% | 2.4 ± 1.5% |
| Midbrain | 5.2 ± 1.8% | 1 ± 0.5% | 2.3 ± 1.2% |
| Inferior colliculi | 5 ± 3.7% | 3 ± 2.3% | 2.6 ± 1.6% |
| Whole brain | 5.2 ± 1.8% | 3.4 ± 1% | 1.9 ± 1.5% |
| Mean | 5.2% | 4.2% | 3.8% |
| SD | 3.3% | 2.8% | 2.4% |

**Table S2**. Multiple unpaired t tests of SUVR of [^18^F]SDM-16 in APP/PS1 and WT mice.


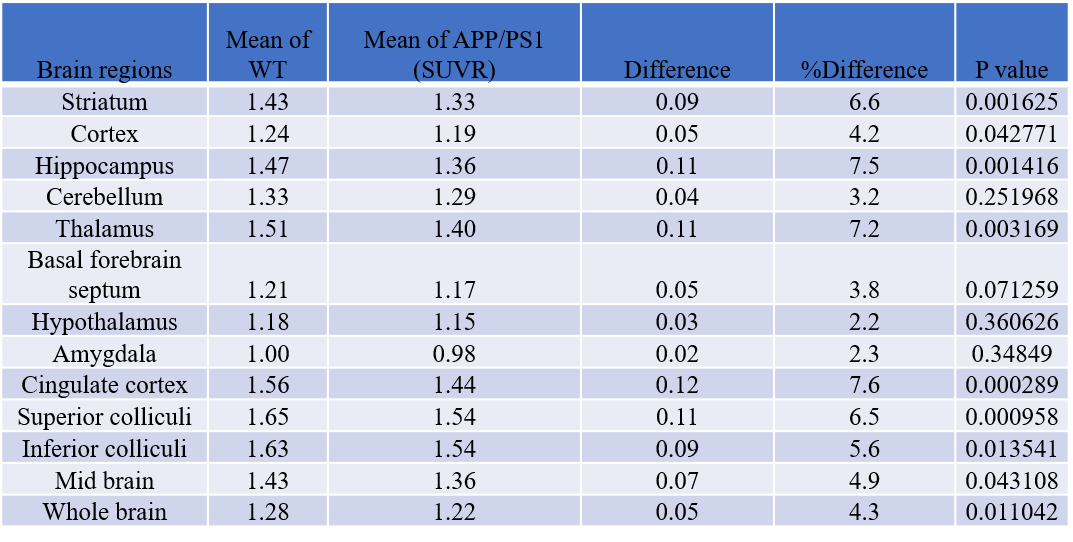


**Table S3**. Multiple unpaired t tests of SUVR of [^11^C]UCB-J in APP/PS1 and WT mice.


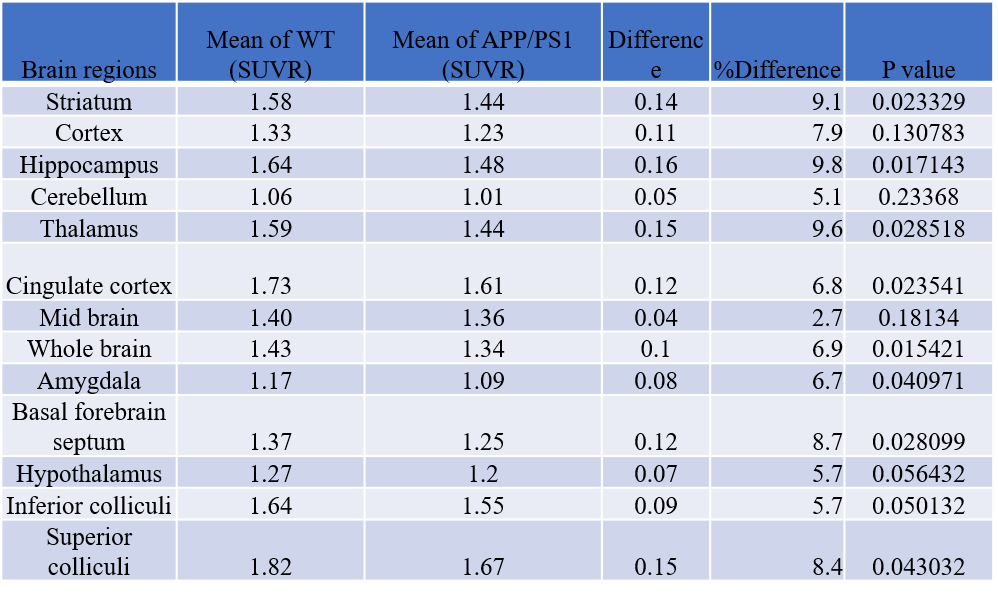


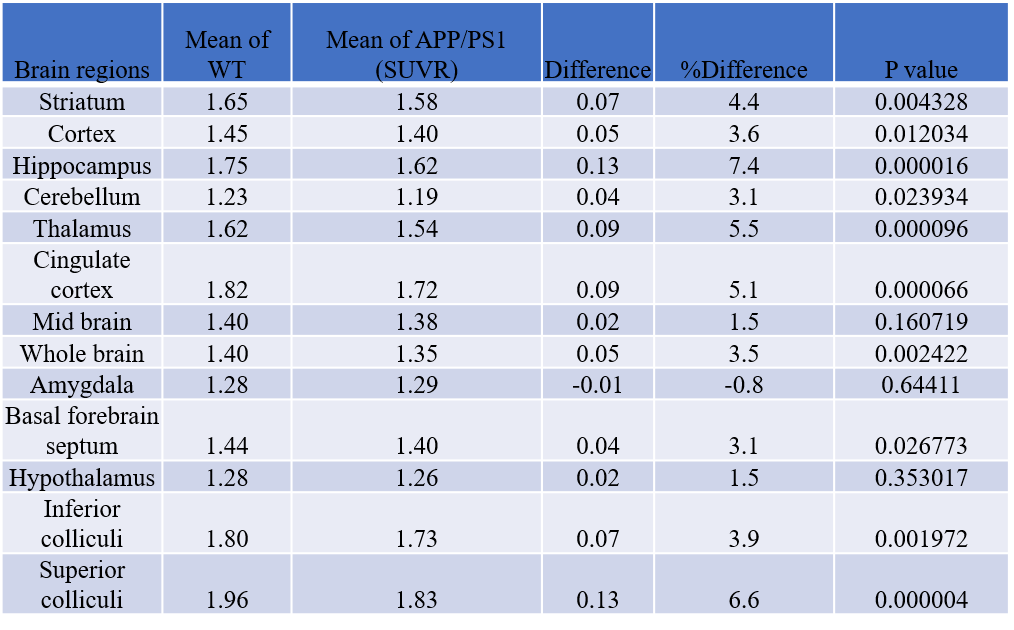
**Table S4**. Multiple unpaired t tests of SUVR of [^18^F]SynVesT-1 in APP/PS1 and WT mice.


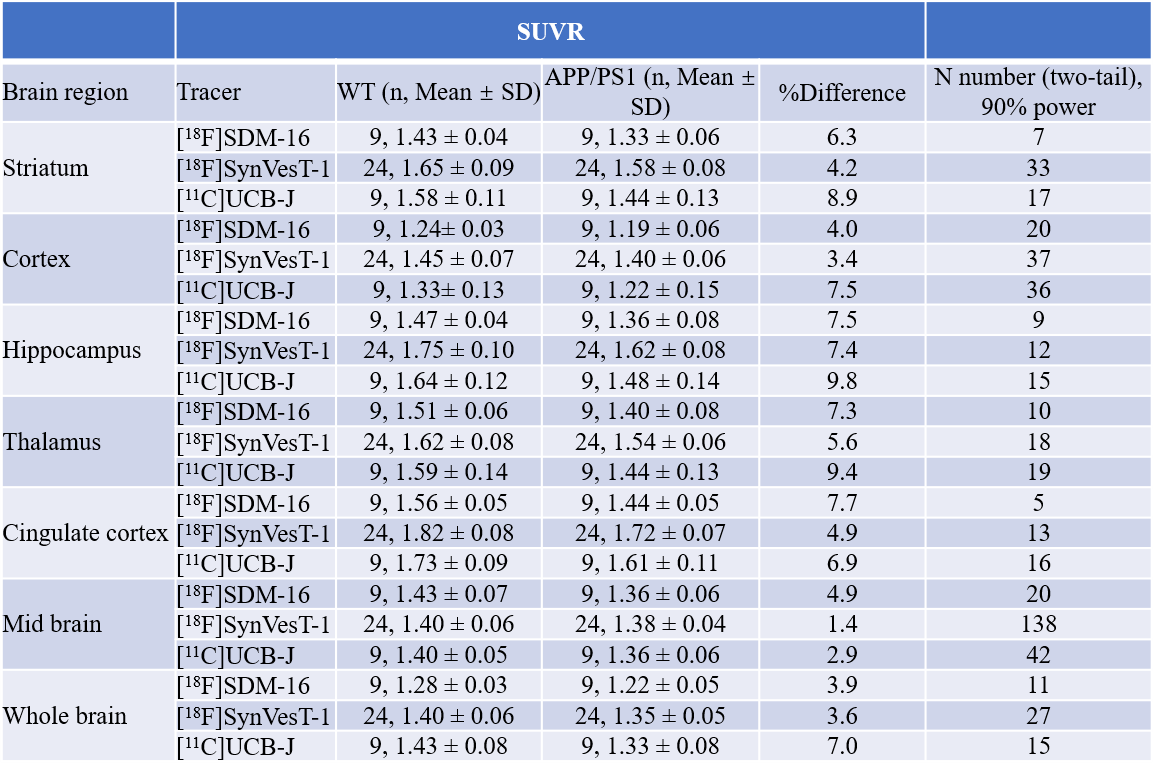
**Table S5**. Sample size calculations based on preliminary mouse brain PET imaging studies on hippocampus and exploratory brain regions using [^18^F]SDM-16, [^11^C]UCB-J, and [^18^F]SynVesT-1.
